# Effects of Combined Training on Visceral Adiposity Index and Metabolic Phenotype in Obesity: A Randomized Clinical Trial

**DOI:** 10.3390/ijerph22091462

**Published:** 2025-09-22

**Authors:** Júlia Elena Fontana Ronsani, Mariana Papini Gabiatti, Anne Ribeiro Streb, Rodrigo Sudatti Delevatti, Giovani Firpo Del Duca, Fernanda Hansen

**Affiliations:** 1 Health Sciences Center—CCS, Department of Nutrition, Federal University of Santa Catarina, Florianopolis 88040-370, Brazil; juliafontanaron@hotmail.com (J.E.F.R.); mpgabiatti@gmail.com (M.P.G.); or fernandahansen@hotmail.com (F.H.); 2 Center of Sports—CDS, Department of Physical Education, Federal University of Santa Catarina, Florianopolis 88040-900, Brazil or anne.streb@ufsc.br (A.R.S.); gfdelduca@gmail.com (G.F.D.D.)

**Keywords:** training periodization, visceral adiposity function, chronic disease, cardiometabolic risk

## Abstract

**Introduction**: The role of obesity in developing metabolic alterations is related to the distribution of adipose tissue, and visceral fat predisposes people to a higher risk than subcutaneous fat. The effect of different forms of periodization of combined training is still unknown in reducing cardiometabolic risk in adults with obesity. This randomized clinical trial aims to compare the effects of 16 weeks of periodized combined training with fixed and linear increase intensities on individuals with obesity, using the visceral adiposity index (VAI) and metabolic phenotype. **Methods**: In total, 59 adults with obesity (61.0% female) were allocated into three groups: control (CG, 34.4 ± 6.9 years; BMI, 33.0 ± 2.5 kg.m^−2^), combined training with fixed intensity (FG, 33.6 ± 8.4 years; BMI, 32.9 ± 2.3 kg.m^−2^), and linear increase intensity (LG, 34.5 ± 6.0 years; BMI, 33.4 ± 2.8 kg.m^−2^) in a 1:1:1 ratio. VAI equations were used with waist circumference, triglycerides, BMI, and HDL-c. The metabolic phenotype was defined by the presence of >3 abnormalities of the following: systolic/diastolic blood pressure, triglycerides, HDL-c, fasting blood glucose, and waist circumference, classified as metabolically healthy and unhealthy (MHO; MUO). Intra- and intergroup analyses were performed per protocol (PP) and intention-to-treat (ITT) using the Generalized Estimated Equations method. *p* < 0.10 was the level of significance adopted for interaction, and *p* < 0.05 was the level of significance for the isolated effect of time and/or group. **Results**: VAI decreased in FG (*p* < 0.001) in PP and ITT analyses, but not in LG in either analysis (*p* > 0.05). There was a higher number of MUO in FG compared to LG, only in PP, considering the effect of group analysis (*p* < 0.01), but not of time or group * time or ITT analyses (*p* > 0.05). **Conclusions**: Combined training with fixed intensity improved VAI but was insufficient to affect metabolic phenotype. These findings suggest minimal differences between fixed intensity and linear increase protocols in reducing the risk of metabolic complications during obesity treatment.

## 1. Introduction

Obesity is characterized by excess body fat that keeps the body in a state of chronic low-grade inflammation [1]. It is a known risk factor for the emergence of new chronic non-communicable diseases (NCDs) [2,3,4]. However, the clinical impact of obesity is not uniform; variations in body fat distribution and adipose tissue function lead to different cardiometabolic health-related outcomes [5,6]. Consequently, some individuals who are overweight may have a metabolic profile similar to those of normal weight [7,8,9].

In visceral obesity, there is a greater accumulation of fat in the abdominal region and a higher level of adipose tissue dysfunction, as fat begins to accumulate in lean tissues [3,10], which is associated with a higher cardiometabolic risk [7]. Consequently, there is increased production of adipokines, pro-inflammatory activity [11,12], and reduced insulin sensitivity [13], increasing the risk of developing type 2 diabetes, cardiovascular diseases, cancer, stroke, and dyslipidemia [14], negatively affecting the quality of life [4]. In contrast, peripheral obesity is characterized by fat deposition in the hip and thigh area, which is associated with a less dysfunctional metabolic profile [9,15]. Therefore, from a clinical perspective, assessing not only body fat but also the adipose tissue distribution becomes essential to evaluate the level of health impairment [6] and to individualize health care, particularly in individuals with obesity. Hence, the visceral adiposity index (VAI) was developed to estimate the functionality of adipose tissue associated with cardiometabolic risk [16,17]. The VAI sex-specific mathematical formula combines anthropometric measurements with biochemical parameters, providing a more sensitive indicator of metabolic health when compared to isolated parameters [8]. Furthermore, being overweight does not necessarily indicate that the individual manifests cardiometabolic changes [15]. Thus, classifying individuals with obesity according to their metabolic phenotype, metabolically healthy obesity (MHO) or metabolically unhealthy obesity (MUO), allows screening of individuals at risk [18]. 

Physical exercise, more specifically, combined training with aerobic and strength exercises in the same session, is effective for weight loss and, consequently, for managing comorbidities associated with obesity [19,20]. Training periodization, in turn, refers to the manipulation of training load components—volume and intensity [21]—and its application is suggested to optimize the health benefits of physical exercise practice [22]. Among the types of periodization, a linear increase involves the progressive overload of volume and/or intensity, focusing on the gradual increase of a variable; and fixed intensity maintains a constant intensity while varying stimuli during training cycles, manipulating variables such as exercises, volume, and training adaptations on a daily, weekly, or biweekly frequency.

Linear periodization has been suggested to be more beneficial than fixed periodization for physical fitness in adults with obesity [23]. Nonetheless, the effect of different forms of periodization of combined training in reducing cardiometabolic risk in adults with obesity is still unknown. This knowledge gap is particularly relevant given the growing prevalence of obesity and the urgent need for strategies to increase exercise adherence to mitigate cardiometabolic risk in this population. Due to the association of increased cardiometabolic risk with visceral adiposity and dysfunctional adipose tissue, investigating the effects of different types of combined training interventions on VAI and metabolic phenotype can provide evidence to support training prescriptions tailored to metabolic health rather than just body weight. However, to date, no clinical trials evaluating the effects of interventions with combined training on the VAI or metabolic phenotype classification have been found. Therefore, the present study aimed to evaluate the effect of combined training with fixed intensity and with linear increase on VAI and metabolic phenotype in adults with obesity.

## 2. Materials and Methods

### 2.1. Experimental Approach to the Problem

This single-blinded randomized controlled trial allocated adults with obesity into three groups: control (CG), combined training with fixed intensity (FG), or linear increase (LG). The study design involved exercise training, so sample blinding was not possible. The subjects knew that they were in the training group and that there was a control group; however, the subjects did not know which training group they were allocated. However, the evaluators were blinded to group allocation. Waist circumference (WC), triglycerides (TGs), body mass index (BMI), HDL-c, systolic/diastolic blood pressure, and fasting blood glucose were evaluated before the intervention and after 16 weeks of training to calculate the VAI and classify the subjects as MHO or MUO. This is a study of the secondary outcomes of a clinical trial [24].

### 2.2. Subjects

The study sample consisted of adult individuals, aged 20 to 50 years, of both sexes, diagnosed with obesity grades I (BMI ≥ 30 kg/m^2^ and ≤34.5 kg/m^2^) and II (BMI ≥ 35 kg/m^2^ and ≤39.5 kg/m^2^). This study was approved by the Ethics and Research Committee with Human Beings of the Federal University of Santa Catarina (UFSC), under protocol number 2,448,674, and published in the Brazilian Registry of Clinical Trials (ReBEC) under the number RBR-3c7rt3. The intervention occurred between June and September 2018, and all participants signed the Free and Informed Consent Form, volunteering to participate in the research.

The exclusion criteria consisted of having regular participation in a physical exercise program (frequency > 3 times a week) in the last three months, being a smoker or former smoker for less than six months, consuming alcohol excessively (≥7 drinks per week for female and ≥14 drinks per week for male), having bone, muscle, or joint lesions limiting the practice of physical exercises, using medications to control/treat obesity, having undergone surgical procedures aiming at weight reduction, and, for women, being in menopause. Additionally, for this study, individuals with TG values > 3.15 mmol/L (>279 mg/dL) or BMI > 40 kg/m^2^, as criteria for estimating VAI, were excluded. A 3-day dietary recall was used as dietary control, and the volume of physical activity and the time spent on sedentary activities before and after the intervention were controlled for any potential interference of these behaviors on the results (which did not occur) [25]. None of the female participants was in menopause. Participants were selected through intentional non-probabilistic sampling. More details about the sampling procedure or other information can be found in the publications of the study protocol [24,25,26].

### 2.3. Intervention Groups

Study participants were randomly allocated into three groups: one control (CG) and two groups of combined training (FG—fixed intensity and LG—linear increase). To balance the proportion of individuals between groups and the characteristics of the participants, randomization stratified by sex, age, and BMI was adopted, with a 1:1:1 ratio. The randomization process was performed in the online software randomizer.org by researchers not involved in the intervention. The allocation list was hidden from all study evaluators, and participants in FG and LG did not know which group they were allocated. 

The period of intervention was 16 weeks. No intervention was applied in the CG. In the intervention groups (FG and LG), all stages of the training protocols were supervised by Physical Education professionals. The first week consisted of familiarization with the training routine, followed by 15 weeks of training protocols. In the first week, the sessions lasted 30 minutes, with the initial half reserved for low-intensity aerobic exercise (30 to 39% reserve heart rate (HRres)) and the final half for strength training, where 6 exercises (the same as the intervention) were performed with only one set each of 10–15 maximum repetitions (RMs). The 15 weeks of intervention lasted one hour. The first 30 minutes were for aerobic training (walking and/or running on an outdoor track), and the final 30 minutes were for strength training (six exercises involving large muscle groups: bench press, leg press, pull down, crucifix machine, free squat, and low row). The intervention groups received training protocols, equivalent in total volume (all intervention period), varying between the types of periodization. Periodic reassessments were performed to adjust the training intensity at each mesocycle. The FG remained, throughout the 15 weeks, at moderate intensity; that is, it maintained aerobic training at moderate intensity (50 to 59% HRres), as well as strength training, with six exercises of two sets of 10 to 12 RMs throughout the time. The LG had a training model with a linear increase in intensity, consisting of three five-week mesocycles. In the first mesocycle, light intensity was used for aerobic training (40 to 49% HRres) and strength training, with six exercises of two sets of 12 to 14 RMs. The second mesocycle progressed to moderate intensity (50 to 59% HRres) and to strength training, from 10 to 12 RMs. In the final mesocycle, participants achieved the highest intensity (60 to 69% HRres) and performed strength training from 8 to 10 RMs. More details on the intervention design with the training protocols of each group can be found in the publication of the clinical trial protocol [24].

### 2.4. Characterization and Anthropometric Parameters

The variables of sample characterization: age (in full years), sex (female and male), and skin color (white and non-white) were collected through the Question Pro platform.

VAI was estimated according to sex [16], using the following variables: WC in cm, BMI in kg/m^2^, TG in mmol/L, and HDL-c in mmol/L, according to the formulas below:

VAI (male) = [WC/(39.68 + 1.88 × BMI)] × (TG/1.03) × (1.31/HDL-c)(1)

VAI (female) = [WC/(36.58 + 1.89 × BMI)] × (TG/0.81) × (1.52/HDL-c)(2)

The classification of metabolic phenotype considered the five indicators present in the "National Cholesterol Education Program's Adult Treatment Panel III" (NCEP-ATP III) [12]. The MUO phenotype was classified according to the presence of three or more inadequacies in the parameters of (i) high blood pressure (≥130 mmHg and/or ≥85 mmHg), (ii) TG (>150 mg/dL), (iii) HDL-c (<40 mg/dL), (iv) fasting blood glucose (>99 mg/dL), and (v) WC (>102 cm and >88 cm for male and female, respectively). 

To measure the BMI of each individual, an electronic scale (Welmy, model W300A, Santa Bárbara d'Oeste, SP, Brazil) with a coupled vertical anthropometer was used. WC was measured in cm at the midpoint between the last rib and the iliac crest.

### 2.5. Clinical and Biochemical Parameters

A portable automatic monitor (Omron®, HEM 742-E, Bannockburn, Illinois, USA) was used for systolic and diastolic blood pressure measurements. Participants were instructed to avoid any consumption of stimulants or alcoholic beverages. Five minutes before the test, participants were instructed to rest supine. Three measurements were made, with an interval of one minute between them, and the mean value between measurements, expressed in mmHg, was considered a reference value. 

Blood samples were collected in the morning. Participants were instructed to fast for 12 hours and not exercise for 72 hours before collection. The collection was performed by venipuncture, with 20 mL of blood collected in vacuum-dried tubes. Samples were processed, centrifuged to obtain serum, and stored in a biofreezer at −80 °C. The manufacturer's instructions were followed for all analyses. From the serum, HDL-c (accelerator selective detergent methodology) and TG (enzymatic colorimetric method (Trinder)) were dosed, both in the BSA20 apparatus, Mindray. An enzymatic colorimetric kit (Labtest Diagnóstica SA, Minas Gerais, Brazil) was used to determine fasting blood glucose values.

### 2.6. Statistical Analysis

The Shapiro–Wilk test verified the symmetry of the data. For the descriptive analysis of the data, mean, standard deviation (SD), and absolute and relative frequency were used. At baseline, differences between groups were tested using one-way ANOVA and Pearson's chi-squared test for continuous and categorical variables, respectively. 

The outcomes were analyzed by per protocol (PP) and by intention-to-treat (ITT) analysis. PP considered data from those who participated until the end of the study, while ITT considered data from all randomized participants, with missing data handled using the multiple imputation method, where each missing value was replaced by the mean of five plausible imputed values. The intra- and intergroup analyses were performed by the method of Generalized Estimated Equations (GEE), adopting the type of linear model for the continuous variable (VAI) and the negative binomial model for the categorical variable (metabolic phenotype). Finally, the Bonferroni post hoc test was performed. The level of significance adopted for interaction was *p* < 0.10, while the isolated effect of time and/or group was *p* < 0.05. Cohen’s *d* was used to estimate effect sizes based on the difference between pre- and post-intervention means in each group. For paired samples, pooled standard deviations were calculated using standard errors and sample sizes. Effect sizes were classified as small (≈0.2), moderate (≈0.5), or large (≥0.8). All analyses were performed using the statistical software IBM SPSS version 21.0 (IBM Corp., Armonk, NY, USA).

## 3. Results

### 3.1. Sample Characterization

**Figure 1:**
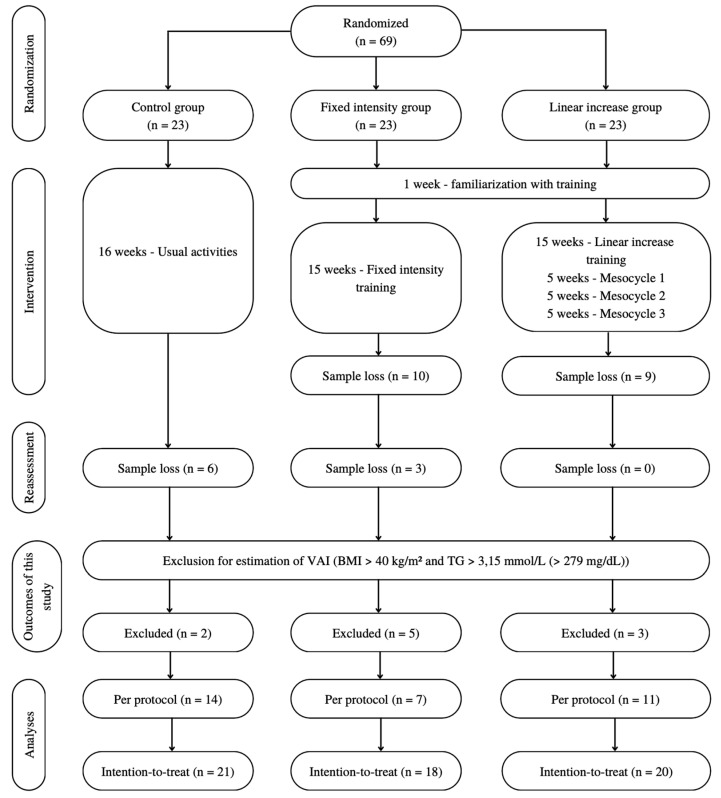
Flowchart of participation of individuals throughout the study.

The initial sample of the clinical trial consisted of 69 participants. In the present study, 10 participants were excluded as criteria for VAI estimation, because they presented BMI > 40 kg/m^2^ and/or TG > 3.15 mmol/L (>279 mg/dL) (Figure 1). The final samples consisted of 32 participants in PP and 59 in ITT analysis. The reasons for dropouts have been previously described [26], and they were handled through ITT analysis.

Therefore, the total number of participants included in this study was 59 (Table 1) (CG (n = 21), FG (n = 18), LG (n = 20)). Of these, 36 (61.0%) were female and 47 (79.7%) had white skin color. In total, 12 individuals (20.3%) were classified with the MUO phenotype. At the baseline, the groups were similar in characterization, anthropometric, clinical, and biochemical parameters, including VAI mean, and the proportion of individuals classified as MUO (*p* > 0.05). The total number of individuals who stayed until the end of the study was 32.

### 3.2. Effects of Combined Training on Visceral Adiposity Index and Metabolic Phenotype

Table 2 shows the results of the analyses performed by PP (n = 32) and by ITT (n = 59) of the VAI by group in the pre- and post-intervention periods. A significant difference was observed in the group * time interaction. It was verified that the intervention with physical training for 16 weeks decreased the VAI, with a moderate effect size, in FG in the analysis by PP (Bonferroni post hoc, *p* < 0.001; Cohen's *d* 0.56) and ITT (Bonferroni post hoc, *p* < 0.001; Cohen's *d* 0.50). For the LG, VAI did not significantly decrease in the PP (*p* > 0.05; Cohen's *d*: 0.03) and ITT analysis (*p* > 0.05; Cohen’s *d*: 0.18).

Table 3 shows the results of the analysis of metabolic phenotypes by group in the pre- and post-intervention periods. In the analyses by PP and ITT, no statistically significant differences were observed in the classification of metabolic phenotype when considering the effect of time and interaction (group * time). However, in the analysis by PP, a significant difference was observed in the classification of metabolic phenotype by group, with FG showing a higher number of MUO individuals from LG post-intervention (Bonferroni's post hoc, *p* = 0.024).

## 4. Discussion

The intervention with combined training for 16 weeks effectively decreased the value of VAI in FG but not in LG. Despite this, both protocols were ineffective in changing the classification from an MUO to MHO phenotype. These findings suggest that, while FG had some impact on VAI, clinically, since neither changed the metabolic phenotype, the overall differences between the protocols are minimal. 

In the present study, the metabolic phenotype is defined by the presence of three or more cardiometabolic alterations [12]. Even if there is improvement in the parameters used to classify the MUO phenotype, if they do not reach the normal/desirable range, the phenotype classification cannot be changed. On the other hand, VAI results from a mathematical calculation that includes four variables, one of which is BMI. Therefore, it may be more sensitive to changes in metabolic health over time. However, there are still no well-established and validated VAI cut-off points for the Brazilian population. Thus, this study only evaluated the impact of the intervention on the VAI value.

When analyzing the mean difference in the raw VAI values from pre- to post-intervention, an increase in the CG was observed (small effect size in both PP, Cohen's *d*: 0.40 and ITT, Cohen's *d*: 0.24), while a decrease in the FG was observed (moderate effect size in both PP, Cohen's *d*: 0.56 and ITT, Cohen's *d*: 0.50). While in the LG, VAI showed a minimal non-significant difference in the PP (Cohen's *d*: 0.03) and in the ITT analysis (small effect size, Cohen's *d*: 0.18), suggesting that LG may have prevented the worsening of the VAI, while FG may have improved its values. 

In the study by Ho et al., 2012 [27], aerobic, resistance, or combined training protocols were compared for 12 weeks in adults with overweight and obesity aged 40 to 66 years. Anthropometric measurements, particularly BMI, WC, and visceral fat (measured using dual-energy X-ray absorptiometry), were significantly lower in the combined training group than in the other groups. This corroborates our FG results, since BMI and WC are parameters used to calculate VAI. Amato and Giordano, 2014 [17], developed the VAI as a formula to validate an adipose tissue distribution model by comparing the values found with results of magnetic resonance imaging (considered the gold standard to differentiate visceral fat from subcutaneous fat) [8,9,28]. VAI was directly correlated with visceral fat. Moreover, the calculation of VAI was applied to 1498 patients in primary health care, and it was observed that VAI values increased as the severity of metabolic complications increased [28]. Thus, VAI becomes a viable measure for use in clinical practice, as it uses routine tests (TG, HDL-c) and simple anthropometric measures (WC, BMI). This makes VAI easy to apply and low-cost, unlike magnetic resonance imaging exams, which require extremely expensive equipment. 

However, while the FG protocol showed a decrease, LG showed only a minimal and non-significant difference in VAI values. At baseline, the mean value of VAI in the FG was 2.59, and in the LG, it was 1.47, which could make short-term improvement more noticeable. However, the baseline VAI or parameters that compose its formula did not differ between groups. Therefore, this may indicate that, in obesity treatment, the magnitude of weight loss and improvement of metabolic health indicators depends not only on the intensity but also on maintaining a higher intensity. In this context, Slentz et al. [29] compared the effects of different volumes and intensities of physical training for eight months on anthropometric parameters in overweight individuals. The authors observed a dose-dependent relationship; that is, higher levels of physical exercise are related to greater improvement in anthropometric parameters—weight loss, body fat loss, and decreased WC—compared to lower levels of exercise. This corroborates our findings, since in our study, besides both groups having the same training load over the 16 weeks of intervention, the LG trained at a lower intensity for five weeks, possibly impacting the observed effects in VAI. Taken together, although baseline VAI values may have influenced the magnitude of change, we consider that the longer exposure to higher training intensity in the FG group was the main factor driving the more pronounced reduction in VAI. 

In the PP analysis of phenotype classification, only FG and LG are statistically different. No significant differences were found in the ITT analysis. When analyzing the raw data, it can be seen that this difference may be related to the fact that there are more individuals with the MUO phenotype classification in both FG and LG compared to CG; at the end of the intervention, there are more individuals with the MUO phenotype in the CG than in the FG and LG, indicating a possible positive effect of combined training on phenotype classification. Nevertheless, this did not occur in the ITT analysis. 

A prospective cohort study by Martinez-Gomez et al., [30] followed more than 200,000 adults in Taiwan for six years (38.5 ± 12.1 years; 50.1% males), where physical activity intensity, frequency, and duration were self-reported and classified as inactive, lower insufficiently active, upper insufficiently active, active, or highly active. The authors observed that individuals with higher self-reported levels of physical activity had a higher probability of converting from the MUO to the MHO phenotype, suggesting a dose-dependent effect of physical activity. Moreover, higher levels of physical activity were associated with a lower risk of developing metabolically unhealthy phenotypes among non-obese individuals. Considering this, the lack of results regarding the conversion of the metabolic phenotype in our study is possibly due to the intervention time and low weekly training frequency. While Martinez-Gomez et al. [30] observed beneficial effects of the physical activity level after several years of cohort follow-up, our 16-week intervention period may not have provided enough time to normalize the parameters considered in classifying metabolic phenotypes. 

Limitations of our study include the small sample size, short intervention time, and participant dropouts, which we handled through imputation in ITT analysis. Also, there was low adherence of the participants of the three groups to the training program [26], and the mean training days per week was 1.6 when the initially proposed was three days. Limitations also included the lack of well-established and validated VAI cut-off points for the Brazilian population. As strengths, we highlight the control of aerobic training variables, with the maintenance of relative intensity in the FG and the gradual increase in intensity in the LG along the mesocycles, both adjusted with HRres.

The study design, with the progression of intensity and similarity in training volume, allows for verifying the effects of the different forms of periodization of the combined training program in an equalized way. Furthermore, this study includes a population with similar characteristics, with all individuals with obesity. Considering that sedentary behavior increases the risk of developing NCDs, combining aerobic and strength exercises is a way to promote health and prevent various diseases, especially NCDs, creating a positive impact on quality of life and also helping with the treatment when diseases are already established [19,20,22]; however, in these populations, based on the results of this clinical trial, it is assumed that the main limitation is the individuals' adherence and commitment to a long-term training program.

## 5. Conclusions

The present study suggests that combined exercise training with fixed intensity for 16 weeks positively affected the parameters that make up the VAI, reducing the value of this index and the risk of metabolic complications in adults of both sexes with obesity. However, these parameter alterations in the 16-week intervention were insufficient to change the MUO phenotype classification to MHO. Taken together, these findings suggest minimal differences between fixed intensity and linear increase protocols in reducing the risk of metabolic complications during obesity treatment.

## Figures and Tables

**Table 1:** Characterization of the sample at baseline (n = 59).

Variables	CG (n = 21)	FG (n = 18)	LG (n = 20)	*p*-Value
	Mean (± SD)	Mean (± SD)	Mean (± SD)	
Age (years)	34.38 (± 6.88)	33.61 (± 8.40)	34.50 (± 6.04)	0.917
BMI (kg/m^2^)	33.01 (± 2.52)	32.92 (± 2.33)	33.41 (± 2.81)	0.819
SBP (mmHg)	116.97 (± 11.38)	120.48 (± 18.64)	115.79 (± 10.66)	0.559
DBP (mmHg)	72.06 (± 5.38)	72.03 (± 8.31)	72.77 (± 6.01)	0.922
TG (mg/dL)	127.28 (± 47.10)	124.77 (± 59.74)	104.60 (± 46.03)	0.311
HDL-c (mg/dL)	58.23 (± 18.77)	54.88 (± 11.92)	55.15 (± 14.72)	0.750
Fasting blood glucose (mg/dL)	100.14 (± 21.29)	96.72 (± 9.66)	96.01 (± 12.10)	0.662
WC all (cm)	108.42 (± 8.97)	104.99 (± 7.77)	108.11 (± 10.12)	0.443
WC male (cm)	113.47 (± 7.92)	112.00 (± 4.61)	112.16 (± 7.98)	0.904
WC female (cm)	104.64 (± 8.01)	101.49 (± 6.60)	105.40 (± 10.80)	0.509
VAI of both sexes	1.76 (± 0.86)	1.85 (± 1.25)	1.53 (± 0.81)	0.579
VAI male	2.01 (± 0.25)	1.85 (± 0.42)	1.91 (± 0.29)	0.937
VAI female	1.58 (± 0.26)	1.85 (± 0.40)	1.27 (± 0.21)	0.408
	n (%)	n (%)	n (%)	
Sex				
Female	12 (57.14)	12 (66.67)	12 (60.00)	0.826
Skin color				
White	17 (80.95)	13 (72.22)	17 (85.00)	0.610
Metabolic Phenotype				
MUO	2 (9.52)	5 (27.78)	5 (25.00)	0.301

BMI: body mass index; DBP: diastolic blood pressure; HDL-c: high-density lipoprotein cholesterol; MUO: metabolically unhealthy obesity; n: number of subjects; SBP: systolic blood pressure; SD: standard deviation; TG: triglyceride; VAI: visceral adiposity index; WC: waist circumference.

**Table 2:** Visceral adiposity index (VAI) in the control, fixed intensity, and linear increase groups before and after 16 weeks of intervention.

Group	Pre (Mean ± SE)	Post (Mean ± SE)	Mean Difference	Cohen’s *d*	*p*-Value		
					Group	Time	Group * Time
Per protocol (n = 32)							
CG (n = 14)	1.78 (± 0.24)	2.18 (± 0.30)	0.40	0.40	0.092	0.504	0.003
FG (n = 7)	2.59 (± 0.52)	1.89 (± 0.42) ^a^	−0.70	0.56			
LG (n = 11)	1.47 (± 0.18)	1.49 (± 0.22)	0.02	0.03			
Intention-to-treat (n = 59)							
CG (n = 21)	1.76 (± 0.18)	1.97 (± 0.21)	0.21	0.24	0.487	0.603	0.001
FG (n = 18)	1.85 (± 0.28)	1.32 (± 0.21) ^a^	−0.53	0.50			
LG (n = 20)	1.53 (± 0.17)	1.69 (± 0.22)	0.16	0.18			

CG: Control group; FG: fixed intensity group; LG: linear increase group; SE, standard error; ^a^ statistically different from the baseline (Bonferroni post hoc test, *p* < 0.001).

**Table 3:** Metabolic phenotype in the control, fixed intensity, and linear increase groups before and after 16 weeks of intervention.

Group	MUO		*p*-Value		
	Pre N (%)	Post N (%)	Group	Time	Group * Time
Per protocol (n = 32)					
CG (n = 14) ^a,b^	2 (14.29)	4 (28.57)	0.021	0.239	0.130
FG (n = 7) ^a^	4 (57.14)	3 (42.86)			
LG (n = 11) ^b^	2 (18.18)	1 (9.09)			
Intention-to-treat (n = 59)					
CG (n = 21)	2 (9.52)	5 (23.81)	0.681	0.700	0.126
FG (n = 18)	5 (27.78)	4 (22.22)			
LG (n = 20)	5 (25.00)	4 (20.00)			

CG: Control group; FG: fixed intensity group; LG: linear increase group; MUO: metabolically unhealthy obesity. ^a,b^ different letters indicate statistical significance (Bonferroni post hoc test, *p* < 0.05).

## Data Availability

The datasets generated and/or analyzed during the current study are available from the corresponding author on reasonable request.

## Abbreviations

The following abbreviations are used in this manuscript:

NCDs	Chronic Non-Communicable Diseases
WHO	World Health Organization
VAI	Visceral Adiposity Index
MHO	Metabolically Healthy Obesity
MUO	Metabolically Unhealthy Obesity
CG	Control Group
FG	Combined Training with Fixed Intensity Group
LG	Combined Training with Linear Increase Group
WC	Waist Circumference
TGs	Triglycerides
BMI	Body Mass Index
HRres	Reserve Heart Rate
RMs	Maximum Repetitions
SD	Standard Deviation
PP	Per Protocol
ITT	Intention-To-Treat
GEE	Generalized Estimated Equation

## References

[1] Khanna D., Welch B.S., Rehman A. (2022). Pathophysiology of Obesity. StatPearls.

[2] Bijari M., Jangjoo S., Emami N., Raji S., Mottaghi M., Moallem R., Jangjoo A., Saberi A. (2021). The Accuracy of Visceral Adiposity Index for the Screening of Metabolic Syndrome: A Systematic Review and Meta-Analysis. Int. J. Endocrinol..

[3] González-Muniesa P., Mártinez-González M.A., Hu F.B., Després J.P., Matsuzawa Y., Loos R.J.F., Moreno L.A., Bray G.A., Martinez J.A. (2017). Obesity. Nat. Rev. Dis. Primers.

[4] Williams E.P., Mesidor M., Winters K., Dubbert P.M., Wyatt S.B. (2015). Overweight and Obesity: Prevalence, Consequences, and Causes of a Growing Public Health Problem. Curr. Obes. Rep..

[5] Goossens G.H. (2017). The Metabolic Phenotype in Obesity: Fat Mass, Body Fat Distribution, and Adipose Tissue Function. Obes. Facts.

[6] Lemieux I., Poirier P., Bergeron J., Alméras N., Lamarche B., Cantin B., Dagenais G.R., Després J.P. (2007). Hypertriglyceridemic Waist: A Useful Screening Phenotype in Preventive Cardiology?. Can. J. Cardiol..

[7] Després J.P. (2006). Is Visceral Obesity the Cause of the Metabolic Syndrome?. Ann. Med..

[8] Kang Y.M., Jung C.H., Cho Y.K., Jang J.E., Hwang J.Y., Kim E.H., Lee W.J., Park J.Y., Kim H.K. (2017). Visceral Adiposity Index Predicts the Conversion of Metabolically Healthy Obesity to an Unhealthy Phenotype. PLoS ONE.

[9] Tchernof A., Després J.P. (2013). Pathophysiology of Human Visceral Obesity: An Update. Physiol. Rev..

[10] Pouliot M.C., Després J.P., Lemieux S., Moorjani S., Bouchard C., Tremblay A., Nadeau A., Lupien P.J. (1994). Waist Circumference and Abdominal Sagittal Diameter: Best Simple Anthropometric Indexes of Abdominal Visceral Adipose Tissue Accumulation and Related Cardiovascular Risk in Men and Women. Am. J. Cardiol..

[11] Brüünsgaard H., Pedersen B.K. (2003). Age-Related Inflammatory Cytokines and Disease. Immunol. Allergy Clin. N. Am..

[12] Lipsy R.J. (2003). The National Cholesterol Education Program Adult Treatment Panel III Guidelines. J. Manag. Care Pharm..

[13] Denino W.F., Tchernof A., Dionne I.J., Toth M.J., Ades P.A., Sites C.K., Poehlman E.T. (2001). Contribution of Abdominal Adiposity to Age-Related Differences in Insulin Sensitivity and Plasma Lipids in Healthy Nonobese Women. Diabetes Care.

[14] Guh D.P., Zhang W., Bansback N., Amarsi Z., Birmingham C.L., Anis A.H. (2009). The Incidence of Co-Morbidities Related to Obesity and Overweight: A Systematic Review and Meta-Analysis. BMC Public Health.

[15] Wildman R.P., Muntner P., Reynolds K., McGinn A.P., Rajpathak S., Wylie-Rosett J., Sowers M.R. (2008). The Obese Without Cardiometabolic Risk Factor Clustering and the Normal Weight with Cardiometabolic Risk Factor Clustering: Prevalence and Correlates of 2 Phenotypes Among the US Population (NHANES 1999–2004). Arch. Intern. Med..

[16] Amato M.C., Giordano C., Galia M., Criscimanna A., Vitabile S., Midiri M., Galluzzo A., Blunda G., Cammisa N., Campo F. (2010). Visceral Adiposity IndexA Reliable Indicator of Visceral Fat Function Associated with Cardiometabolic Risk. Diabetes Care.

[17] Amato M.C., Giordano C. (2014). Visceral Adiposity Index: An Indicator of Adipose Tissue Dysfunction. Int. J. Endocrinol..

[18] Chung H.S., Lee J.S., Song E., Kim J.A., Roh E., Yu J.H., Kim N.H., Yoo H.J., Seo J.A., Kim S.G. (2021). Effect of Metabolic Health and Obesity Phenotype on the Risk of Pancreatic Cancer: A Nationwide Population-Based Cohort Study. Cancer Epidemiol. Biomark. Prev..

[19] Brunelli D.T., Chacon-Mikahil M.P.T., Gáspari A.F., Lopes W.A., Bonganha V., Bonfante I.L.P., Bellotto M.L., Libardi C.A., Cavaglieri C.R. (2015). Combined Training Reduces Subclinical Inflammation in Obese Middle-Age Men. Med. Sci. Sports Exerc..

[20] Schwingshackl L., Dias S., Strasser B., Hoffmann G. (2013). Impact of Different Training Modalities on Anthropometric and Metabolic Characteristics in Overweight/Obese Subjects: A Systematic Review and Network Meta-Analysis. PLoS ONE.

[21] Evans J.W. (2019). Periodized Resistance Training for Enhancing Skeletal Muscle Hypertrophy and Strength: A Mini-Review. Front. Physiol..

[22] Ahmadizad S., Ghorbani S., Ghasemikaram M., Bahmanzadeh M. (2014). Effects of Short-Term Nonperiodized, Linear Periodized and Daily Undulating Periodized Resistance Training on Plasma Adiponectin, Leptin and Insulin Resistance. Clin. Biochem..

[23] Chikih C., Anggunadi A. (2023). Periodization Method of Physical Exercise for Obese People. J. Keolahragaan.

[24] Streb A.R., da Silva R.P., Leonel L., dos S., Tozetto W.R., Gerage A.M., Benedet J., Delevatti R.S., Turnes T., Del Duca G.F. (2019). Comparison of Linear Periodized and Non-Periodized Combined Training in Health Markers and Physical Fitness of Adults with Obesity: Clinical Trial Protocol. Contemp. Clin. Trials Commun..

[25] de Barcelos G.T., Del Duca G.F., de Oliveira Medeiros P.R., Crochemore-Silva I., Gerage A.M. (2020). Effect of Physical Training Periodization on Physical Activity Level in Adults with Obesity. Rev. Bras. Ativ. Fis. Saúde.

[26] Streb A.R., Tozetto W.R., Bertuol C., Benedet J., Del Duca G.F. (2022). Motivos de Adesão, Aderência e Desistência de Adultos Com Obesidade Em Um Programa de Exercícios Físicos. Rev. Bras. Ativ. Fis. Saúde.

[27] Ho S.S., Dhaliwal S.S., Hills A.P., Pal S. (2012). The Effect of 12 Weeks of Aerobic, Resistance or Combination Exercise Training on Cardiovascular Risk Factors in the Overweight and Obese in a Randomized Trial. BMC Public Health.

[28] Amato M.C., Giordano C., Pitrone M., Galluzzo A. (2011). Cut-off Points of the Visceral Adiposity Index (VAI) Identifying a Visceral Adipose Dysfunction Associated with Cardiometabolic Risk in a Caucasian Sicilian Population. Lipids Health Dis..

[29] Slentz C.A., Duscha B.D., Johnson J.L., Ketchum K., Aiken L.B., Samsa G.P., Houmard J.A., Bales C.W., Kraus W.E. (2004). Effects of the Amount of Exercise on Body Weight, Body Composition, and Measures of Central Obesity: STRRIDE—A Randomized Controlled Study. Arch. Intern. Med..

[30] Martinez-Gomez D., Ortega F.B., Hamer M., Lopez-Garcia E., Struijk E., Sadarangani K.P., Lavie C.J., Rodríguez-Artalejo F. (2019). Physical Activity and Risk of Metabolic Phenotypes of Obesity: A Prospective Taiwanese Cohort Study in More Than 200,000 Adults. Mayo Clin. Proc..

